# Biostratinomic alterations of an *Edmontosaurus* “mummy” reveal a pathway for soft tissue preservation without invoking “exceptional conditions”

**DOI:** 10.1371/journal.pone.0275240

**Published:** 2022-10-12

**Authors:** Stephanie K. Drumheller, Clint A. Boyd, Becky M. S. Barnes, Mindy L. Householder

**Affiliations:** 1 Department of Earth and Planetary Sciences, University of Tennessee–Knoxville, Knoxville, Tennessee, United States of America; 2 Fossil Resource Management Program, North Dakota Geological Survey, Bismarck, North Dakota, United States of America; 3 State Historical Society of North Dakota, Bismarck, North Dakota, United States of America; University of Wisconsin Madison, UNITED STATES

## Abstract

Removal or protection from biostratinomic agents of decomposition, such as predators and scavengers, is widely seen as a requirement for high-quality preservation of soft tissues in the fossil record. In this context, extremely rapid burial is an oft-cited mechanism for shielding remains from degradation, but not all fossils fit nicely into this paradigm. Dinosaurian mummies in particular seemingly require two mutually exclusive taphonomic processes to preserve under that framework: desiccation and rapid burial. Here we present a recently prepared *Edmontosaurus* mummy that reveals an alternate fossilization pathway for resistant soft tissues (e.g., skin and nails). While the skin on this specimen is well-preserved in three dimensions and contains biomarkers, it is deflated and marked by the first documented examples of injuries consistent with carnivore activity on dinosaurian soft tissue during the perimortem interval. Incomplete scavenging of the carcass provided a route for the gases, fluids, and microbes associated with decomposition to escape, allowing more durable soft tissues to persist through the weeks to months required for desiccation prior to entombment and fossilization. This pathway is consistent with actualistic observations and explains why dinosaurian skin, while rare, is more commonly preserved than expected if extreme circumstances were required for its preservation. More broadly, our assumptions guide specimen collection and research, and the presence of soft tissues and biomolecules in fossils that demonstrably were not rapidly buried, such as this mummy, suggests that such types of evidence may be substantially more common than previously assumed.

## Introduction

The term “mummy” has become an informal catch-all for fossils exhibiting extremely well-preserved skin and sometimes other soft tissues [1–3] found in isolation rather than within the context of a broader Lagerstätten-style deposit. These mummies are often described as exhibiting evidence of two taphonomic processes that appear to be mutually exclusive: desiccation, which requires long-term residence on the landscape pre-burial [1, 4, 5], and rapid burial, an often imprecisely defined interval assumed to encompass the immediate peri- and postmortem interval [e.g. 3, 6–9]. Rapid burial is argued to hinder advanced decomposition and other biostratinomic processes, especially predation and scavenging [1–2, 4, 10] prior to entombment and subsequent diagenetic alteration of the remains. Explanations for these conflicting preservational pressures are often speculative and unsatisfactory, as they rely on unrealistically rapid modes of desiccation or disregard the effects of smaller scavengers and decomposers, especially invertebrate and microbial communities [3, 6, 10–12].

Previous attempts to characterize the preservation of a natural mummy of the hadrosaurian dinosaur *Edmontosaurus* sp. (NDGS 2000, formerly MRF-03) from the Hell Creek Formation of southwestern North Dakota struggled with the same seemingly conflicting evidence of exceedingly rapid burial and desiccation within a wet paleoenvironment [7, 8]. The specimen preserves large swaths of extremely well-preserved, if desiccated, skin and associated dermal structures (such as keratinous sheaths over the unguals) on the right forelimb, hind limbs, and tail. Historically, soft tissues in such mummies were thought to have decayed away, leaving behind molds or sediment infilled casts of the original soft tissue [2], but molecular sampling of NDGS 2000 yielded putative dinosaurian biomarkers (e.g. degraded proteins, etc.), suggesting the soft tissue was preserved directly in this specimen [13].

Recent preparation of previously concealed anatomical regions of NDGS 2000 revealed patterns of soft tissue damage consistent with injuries caused by predators or scavengers. Here we describe these injuries, the first examples of unhealed carnivore damage in dinosaurian soft tissue, and compare the disposition of the remains to patterns of decomposition and desiccation observed in actualistic contexts. Further, computed tomographic (CT) scanning of regions of the mummy, partnered with grain size analysis of the sediments surrounding the fossil, provide a way to explain the seemingly contradictory phenomena suggested to drive dinosaurian mummy preservation and expand the model of soft tissue preservation to encompass similar examples of natural mummification.

## Materials and methods

### Institutional abbreviations and disposition of the specimen

The partial skeleton of *Edmontosaurus* sp. that forms the focus of this study was collected under permit by and previously reposited within the collections of the Marmarth Research Foundation (MRF) under the catalog number MRF-3 [e.g. 8, 14]. Ownership of this specimen was transferred to the State of North Dakota in 2016 and it now permanently resides in the North Dakota State Fossil Collection under the care of the North Dakota Geological Survey (NDGS) in Bismarck, North Dakota. This specimen is now cataloged under the number NDGS 2000.

### Geologic setting

**Fig 1:**
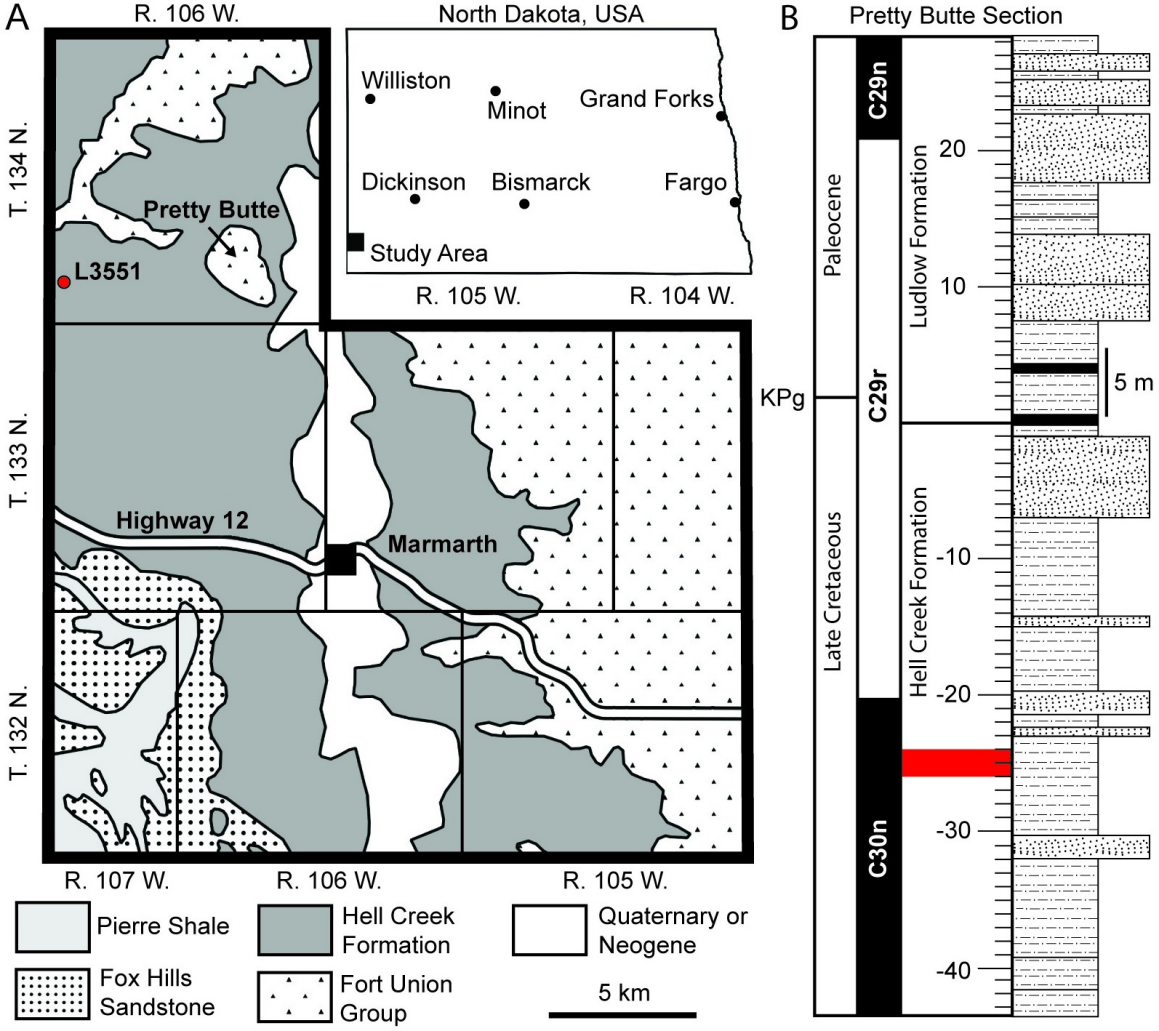
Geographic (A) and stratigraphic (B) position of NDGS 2000. The red dot in (A) indicates the locality where NDGS 2000 was recovered and the red band in (B) indicates the approximate stratigraphic position of NDGS 2000 relative to the published stratigraphic section from nearby Pretty Butte [6: Fig 11]. Geology in (A) based on a digitized version of the map produced by [87]. Position of Cretaceous-Paleogene (KPg) boundary and magnetostratigraphy in (B) from [88]. The KPg boundary and the Hell Creek-Ludlow Formation contact are not coincident at Pretty Butte, as reported by [18].

NDGS 2000 was recovered from locality NDGS L3551 in Slope County, North Dakota within the Hell Creek Formation (Fig 1). The locality where NDGS 2000 was collected preserves mudstones, siltstones, and sandstones. Previous sedimentological and palynological analyses concluded that strata were laid down by fluvial to deltaic deposition within a forested coastal plain ecosystem [8, 14]. While the Hell Creek Formation is perhaps best known for its abundant dinosaurian fossils, it preserves a diverse array of organisms across terrestrial, freshwater, and brackish environments [e.g. 15–18].

The stratigraphic position of NDGS 2000 was previously reported at approximately 60 to 70 meters below the Cretaceous-Paleogene (KPg) boundary [8, 14: Fig 1B]. More recent work at the locality reveals the specimen was situated approximately 25 meters below the KPg boundary [T. Lyson, pers. comm., 2021]. The nearest published stratigraphic section is along the southern portion of Pretty Butte four miles to the east of the locality [6: Fig 11], which is reproduced in Fig 1B with the approximate stratigraphic position of NDGS 2000 indicated in red for reference.

### Specimen preparation

**Fig 2:**
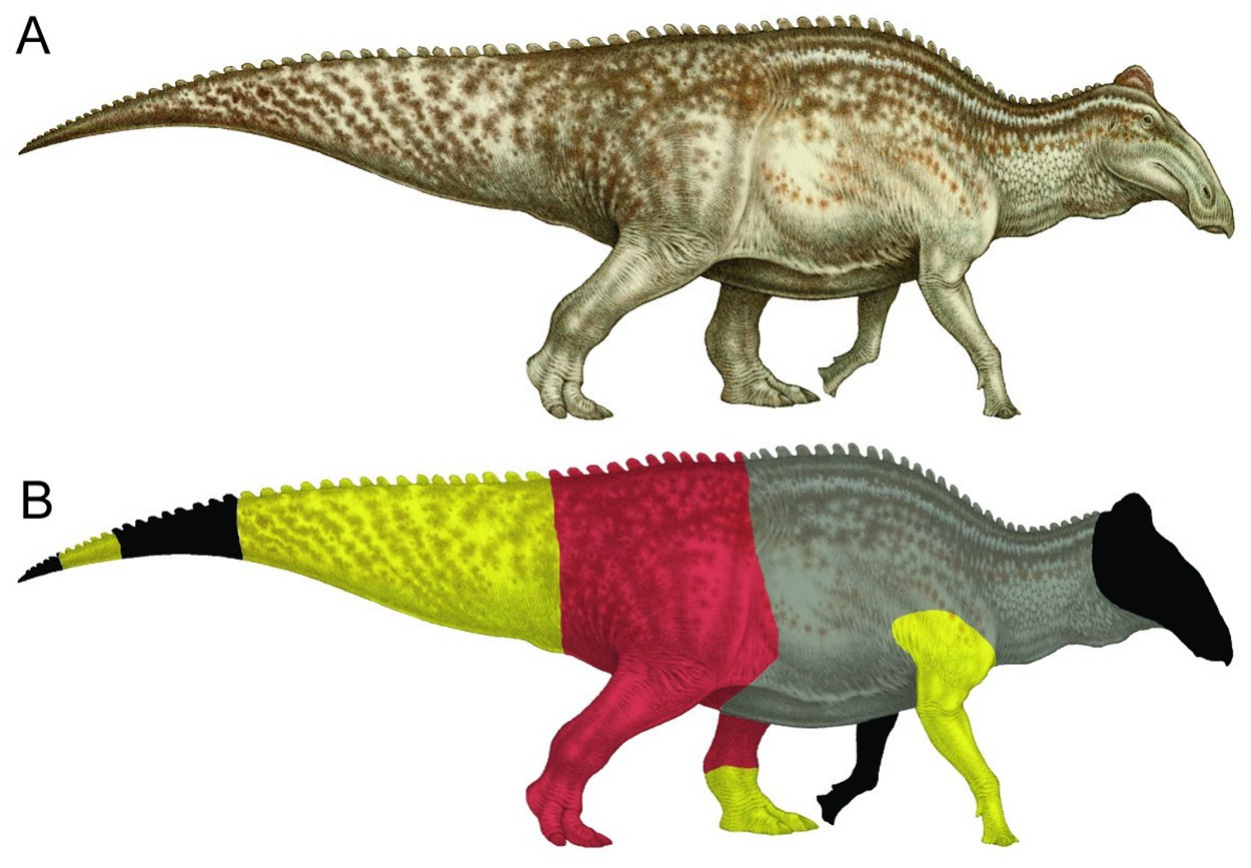
Distribution and current state of preparation of preserved skin on NDGS 2000. (A) Life reconstruction of *Edmontosaurus* sp. based on NDGS 2000 in right lateral view. (B) Areas of skin preservation in NDGS 2000. In (B), black areas indicate portions of the skeleton apparently missing from the specimen, light grey areas indicate regions where the skeleton is preserved but no skin is currently preserved, red areas indicate regions where skin is present and is still undergoing preparation, yellow areas indicate areas where the skin is fully prepared and were examined in this study. Paleoart by Natee Puttapipat.

Preparation of NDGS 2000 under direction of the North Dakota Geological Survey (NDGS) began in February of 2008. Previous preparation was done at other facilities prior to this date, including at the Black Hills Institute of Geological Research in Hill City, South Dakota, but detailed records of that work were not available during this study. While at NDGS, different techniques and tools were used on separate body regions, owing to differences in preservation across the specimen. Magnification (glasses, visors, or magnification lamps) was used for all fine preparation, especially when working close to the skin. The distal-most end of the tail block is preserved in a fine-grained sandstone, where gentle removal of matrix was done using Exacto knives. Some areas only exhibited a slight change in color from the matrix to the fossils that indicated the position of the skin. The caudal portion of the thigh and the anterior-most portion of the tail were better preserved, although still delicate. Pneumatic air scribes (PaleoTools Micro Jack sizes 2 and 3) were used in those areas for matrix removal, working top-down in a repeating crosshatch pattern. That top-down method was used because the uncertain position of the skin owing to rips, folds, tears, and wrinkles made matrix removal moving in from the side of the block impractical and would risk shearing off skin folds. Particle size during removal was very fine, and dust collectors were utilized to remove the loosened matrix. The middle portion of the tail, the feet, and most of the right forelimb were preserved within matrix that was cemented by very dense minerals (e.g., iron oxides). Using pneumatic air scribes (PaleoTools Micro Jack sizes 4 and 5), again working top-down in a repeating crosshatch pattern, the scales in these areas could be “felt” with the point of the tool as a difference in resistance between matrix and fossil. A very gentle hand could allow the tool to float just above the scale, removing all the fine matrix adjacent to the skin. On the right forelimb, more detailed removal of the matrix between the scales was achieved using the smaller PaleoTools Micro Jack size 1. In some body regions (mid-tail, right elbow, feet) the matrix included iron oxide nodules that needed stout tools for removal. In these cases, the larger PaleoARO, ME-9100, and Super-Jack pneumatic tools were used, as well as grinders and rock-saws. These tools were only used when well away from the skin surface. Nodules extended all the way down into the skin in some cases and were left in place in those situations so as to avoid damaging the underlying fossil. In other body regions, the nodules approached the fossil, but a 2–4 mm thick rind of poorly cemented matrix was situated adjacent to the skin, allowing for easier removal of the nodule. In all areas, once a small patch of skin was exposed, preparators worked out from those spots, following the contours of the skin. Having a local frame of reference to where the skin surface was situated allowed faster preparation through the matrix. Where the fossil was cracked, unstable, or broken, repair was done using polyvinyl butyral (B76) dissolved in acetone or an ethyl methacrylate co-polymer (Paraloid B72) dissolved in acetone. In areas where poorly consolidated matrix needed stabilization, polyvinyl butyral (B98) dissolved in ethanol was applied to provide support. Preparation of NDGS 2000 is still in progress in the area containing most of the hind limbs and the pelvic girdle and observations in this study are limited to the completed right forelimb, left pes, and left lateral surface of the tail (Fig 2).

### Feeding trace identification and classification

Classifications for bite marks previously centered on modifications of hard, mineralized tissues, mainly bones [19]. In that context, bite marks are differentiated from other types of bone surface modifications based on the presence of localized impact and crushing damage [19]. The taphonomic classification scheme for such traces captures the relative depth of penetration (indenting cortical bone vs. piercing it) and presence or absence of lateral movement of the tooth during the biting event [19]. We are adapting this terminology for use in soft tissue modifications by using only the more penetrative terms–punctures for tooth marks formed when a tooth penetrates a surface and exhibits no subsequent lateral movement, and furrows for penetrative tooth marks that include a lateral component. Given the more pliable, elastic nature of soft tissue in general, and skin in particular, bites that only indented the surface are not expected to be preserved in the fossil record, and therefore the corresponding terms–pits and scores–will only be used in the context of bone surface modifications. Also, while originally erected to characterize tooth marks, similar terminology has also been used to describe modifications made by claws [20–23] and will be applied in a similar manner in this study.

For bite marks on bone, the basic terminology of Binford [19] can be further modified to reflect specific morphologies of the impacting tooth and how they are reflected in the ensuing bone surface modification. For large-bodied predators most relevant to the Hell Creek ecosystem, serrated teeth, such as those seen in theropod dinosaurs, can leave striated tooth marks, fan- or comb-shaped reflections of denticle size and spacing as an individual tooth drags along a bitten surface [24]. Most (but not all) crocodyliforms lack serrated dentition, and instead have conical, strongly carinated teeth. This morphology is reflected in bite marks as bisections, in which the prominent carina leaves a subscore within the body of a larger tooth mark or notches along the margin of each mark, and these marks are considered diagnostic of a crocodyliform trace maker [25–28]. Theropods and crocodyliforms both exhibit an inertial feeding strategy, in which rapid movements of the head are used to take advantage of acceleration and gravity to reposition prey items in the mouth. This feeding behavior is correlated with bite marks called hook scores, which are J- or L-shaped scores formed when an impacting tooth rapidly changes direction during a single biting event [25–28]. Lastly, when multiple teeth impact a bitten surface during a single biting event, these suites of traces are referred to as serial bite marks [19], and they can be useful hints as to the spacing of teeth and the curvature along the tooth row, when attempting to link traces with potential trace makers [e.g. 29–35].

All marks were measured with length equal to the longest axis of the feature and width the widest point perpendicular to that. For the elongate marks (i.e. furrows) present on the tail, the trajectory of each mark was measured as angles relative to the base of the segmented tail frill [sensu 36] using the image analysis software ImageJ [37]. These measurements were then visualized as rose diagrams [sensu 26] and tested for significance using a Rayleigh’s test of uniformity in the statistical software package PAST 4.07b [38]. Directionality of the initial formation of each injury was not assumed, and so angles were communicated with an axial orientation.

### Sediment analysis

During the early stages of preparation of the tail block and the body block of NDGS 2000 a grid was used to mark several roughly 10 cm by 10 cm squares on the unprepared surface in different regions of the specimen. As preparation work progressed, those marked squares were left intact and pillars of in situ sediment were formed. Once the bone layer was reached those pillars were wrapped in aluminum foil, heavily taped to provide support, and removed from the specimen. Those sediment columns remained sealed and stored for any future studies. As a part of this investigation, the tallest of those sediment pillars (50 centimeters tall), which was collected from under the left femur, was split in half from top to bottom. One half was retained for future studies. The other half was divided into 10 centimeter segments and 500 grams of sediment were removed from each segment, creating five samples, to assess changes in grain size in the rock immediately below NDGS 2000. A sixth sample was collected from within the fossil layer itself adjacent to the in situ sternals. Those six samples were sent to the North Dakota Department of Transportation’s materials testing laboratory for grain size analysis. The encasing rock was also examined by the authors for the presence of sedimentary structures that might reflect the depositional conditions under which NDGS 2000 was entombed. Unfortunately, the fossil locality subsequently has been leveled, preventing more extensive sampling of the fossil-bearing horizon, such as variation in sediment size relative to distance from the remains.

### Computed tomography scans

The distal portion of the right forelimb of NDGS 2000, composed of most of the metacarpals (some of the proximal ends are still attached to the remainder of the specimen) through the unguals of manual digits II through V, is preserved as a single piece separated along a pre-existing fracture from the rest of the forelimb. To better visualize the internal preservation of the fossil, assess whether the flattened appearance of the skin indeed results from extensive desiccation and not postdepositional compression, and search for evidence of additional soft tissues using nondestructive techniques, X-ray computed tomography (CT scanning) was applied to the right forelimb. The scan was conducted at the University of Texas High-Resolution X-ray CT Facility using an NSI scanner with a Fein Focus High Power source (220 kV; 0.125 mA; brass filter; Perkin Elmer detector) via a helical, non-continuous scan. The original CT data set consists of 4612 consecutive slices having an interslice spacing and a uniform voxel size of 99.1 μm. The resulting images were viewed in the program Dragonfly (version 2020.1.1.809). A copy of these scans is available to researchers upon request from the North Dakota Geological Survey.

## Results

### Taphonomy and conditions of the soft tissues

**Fig 3:**
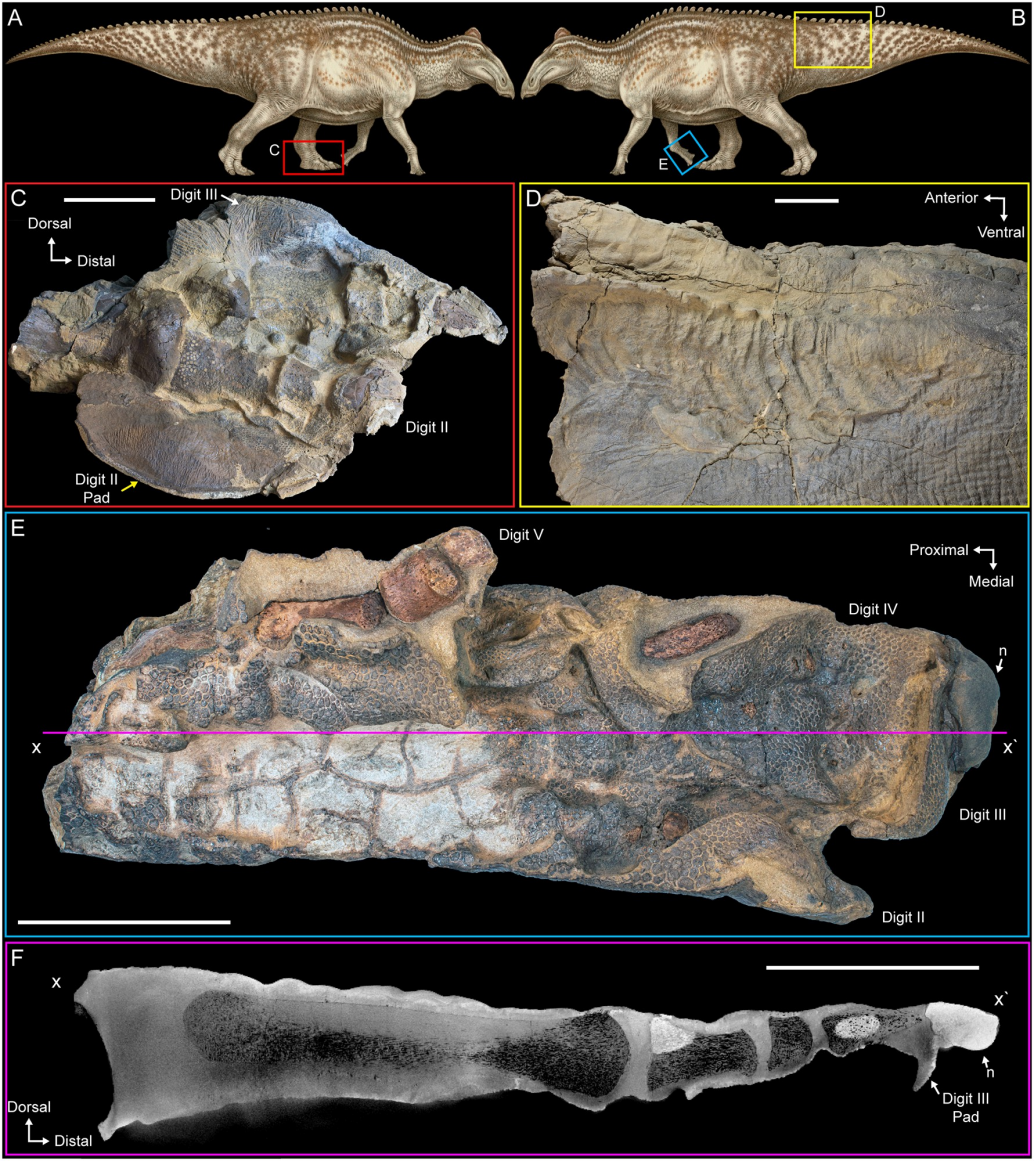
Evidence of desiccation in NDGS 2000. (A) NDGS 2000 reconstruction in right lateral view. (B) NDGS 2000 reconstruction in left lateral view. (C) Left pes in medial view. (D) left lateral view of the proximal portion of the tail. (E) Right manus in ventral view. (F) CT image of cross section of right manus along line x to x’ in (E). Colored boxes and lines in (A), (B), and (E) indicate the locations and views of the photographs in the other figure parts. Paleoart in (A) and (B) by Natee Puttapipat. All scale bars equal 10 cm. Abbreviations: n, iron oxide nodule.

NDGS 2000 is characterized by the strikingly form-fitted, flattened appearance of much of the skin (Fig 3). The tail is 95 cm deep near its base, and this morphology was interpreted as evidence that the specimen had an extremely thick, muscular, or fatty tail in life [39]. Yet the mediolateral width of the tail is extremely thin in places, especially in areas without underlying bones, giving the tail a deflated appearance. This deflation is even more apparent in the left pes, where the texture of the skin is well-preserved, but it lies directly on the underlying bones, and the skin of the toe pads pools adjacent to the bones as largely deflated, flattened structures only a few millimeters in thickness (Fig 3C). In life, these regions of the pes would have included thick, fleshy pads to distribute weight, a reconstruction based on both anatomical and footprint evidence [30, 31, 40–42]. Similar tight association of the skin to the bones is also seen on the ventral side of the right manus (Fig 3E).

**Fig 4:**
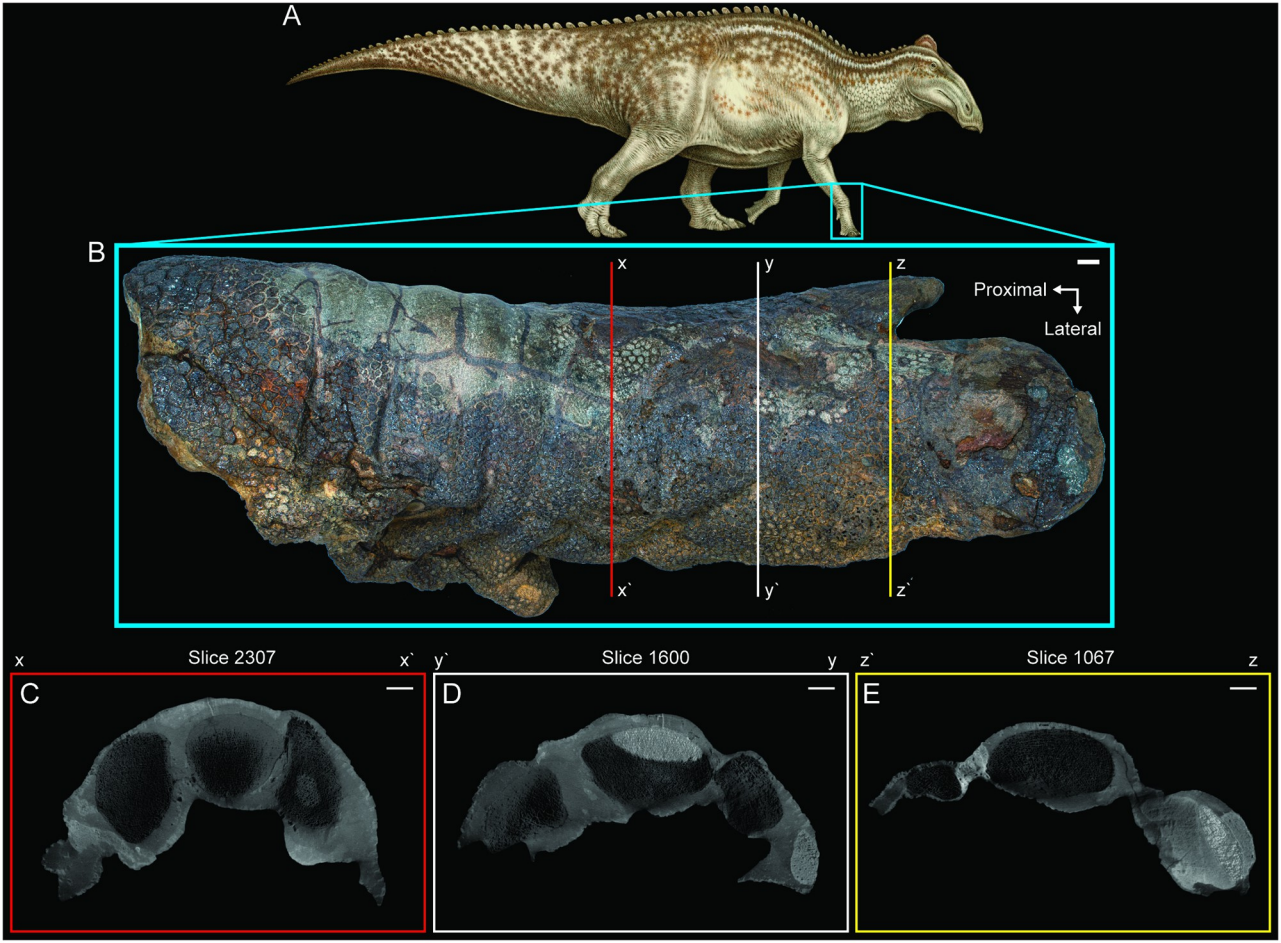
Cross sectional views through the right manus of NDGS 2000. (A) NDGS 2000 reconstruction in right lateral view. (B) Right manus in dorsal view indicating the positions of the three cross sectional views. (C) CT image along line x to x’. (D) CT image along line y to y’. (E) CT image along line z to z’. In (C), (D), and (E), slice numbers from the original CT data are provided above each image. Paleoart in (A) by Natee Puttapipat. Scale bars equal 1 cm.

The CT imaging reveals that the underlying bones of the right manus are undistorted in three dimensions, indicating the observed flattening is not the result of taphonomic compression (Figs 3F and 4). Though the preservation of the skin in particular is exquisite, no underlying tissues other than bones are visible on the CT scans and no visible evidence of their presence was noted during mechanical preparation of the specimen. Instead, the skin is preserved three-dimensionally in extremely close association with the bones, directly overlying them in most regions of the specimen. In short, the fossilized skin is deflated, not compressed, with individual structures in the well-preserved skin and bones largely intact, while the muscles and other internal organs are wholly missing (Figs 3 and 4). These findings are further supported by prior, largely unsuccessful attempts to obtain high resolution CT scans of the tail and body block [7] and more successful sampling of the soft tissues and surrounding sediments for preserved biomolecules, which persist in the mummified skin and fossilized tendons of the specimen but are absent in the rock preserved immediately internal and external to the skin [13, 43, 44].

### Soft tissue and bone surface modifications

**Fig 5:**
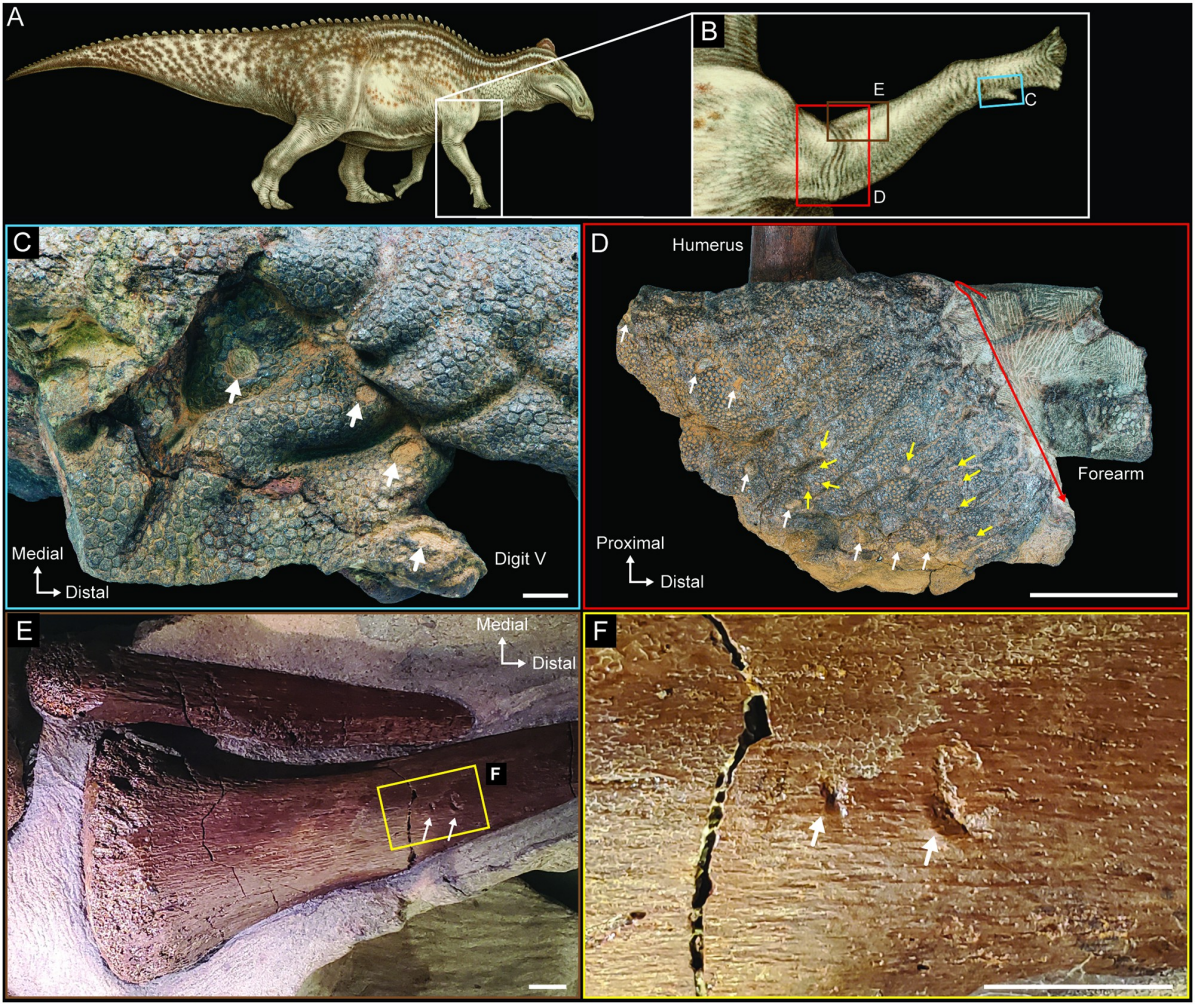
Evidence of soft tissue and bone modifications in NDGS 2000. (A) NDGS 2000 reconstruction in right lateral view. (B) Right forearm in right lateral view indicating the positions of the close-up views in C-H. (C) Dorsal view of manual digit V with white arrows showing a set of punctures in the skin. (D) Right lateral view of right elbow region showing the internal surface of the skin that was degloved from around the right humerus. White arrows highlight positions of larger punctures in the skin, yellow arrows highlight positions of smaller punctures in the skin, and the red arrow indicates the folded edge of the skin flap. (E) Proximal portion of right radius in anterolateral view exposed through a rip in the skin with white arrows highlighting the placement of pits on the bone surface. (F) Close-up view of pits (white arrows) on the right radius shown in (E). Paleoart in (A) by Natee Puttapipat. Scale bar in D equals 10 cm, all other scale bars equal 1 cm.

**Fig 6:**
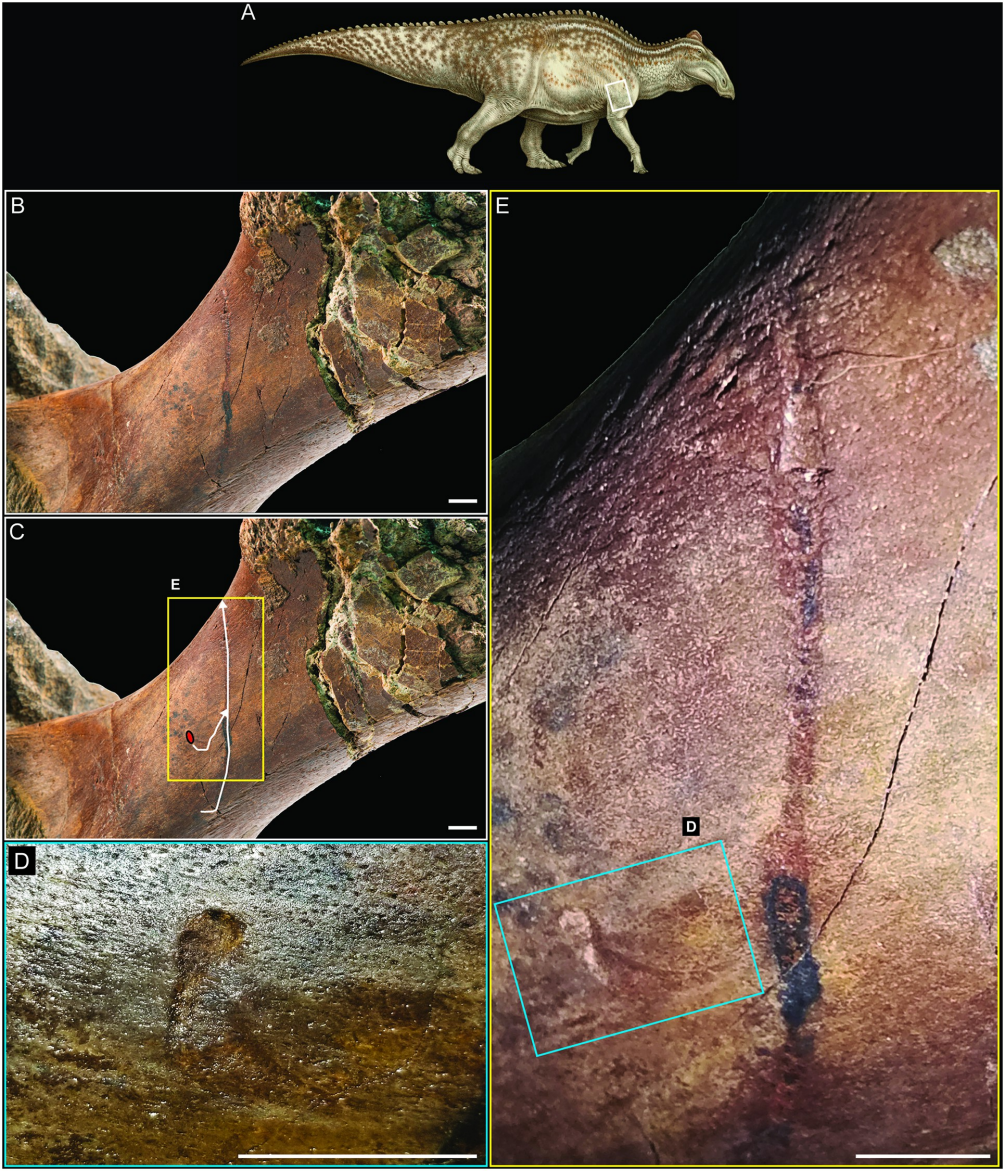
Additional evidence of bone surface modifications in NDGS 2000. (A) NDGS 2000 reconstruction in right lateral view. (B) Right humerus in anterior view denoting the serial hook scores (unmodified image). (C) Right humerus in anterior view showing serial hook scores with red ellipse highlighting the bisected bit and white arrows tracing the hook scores and indicated direction of the tooth across the bone. (D) Close up of bisected pit on the right humerus. (E) Close up of best preserved hook score on the right humerus. All scale bars equal 1 cm.

**Fig 7:**
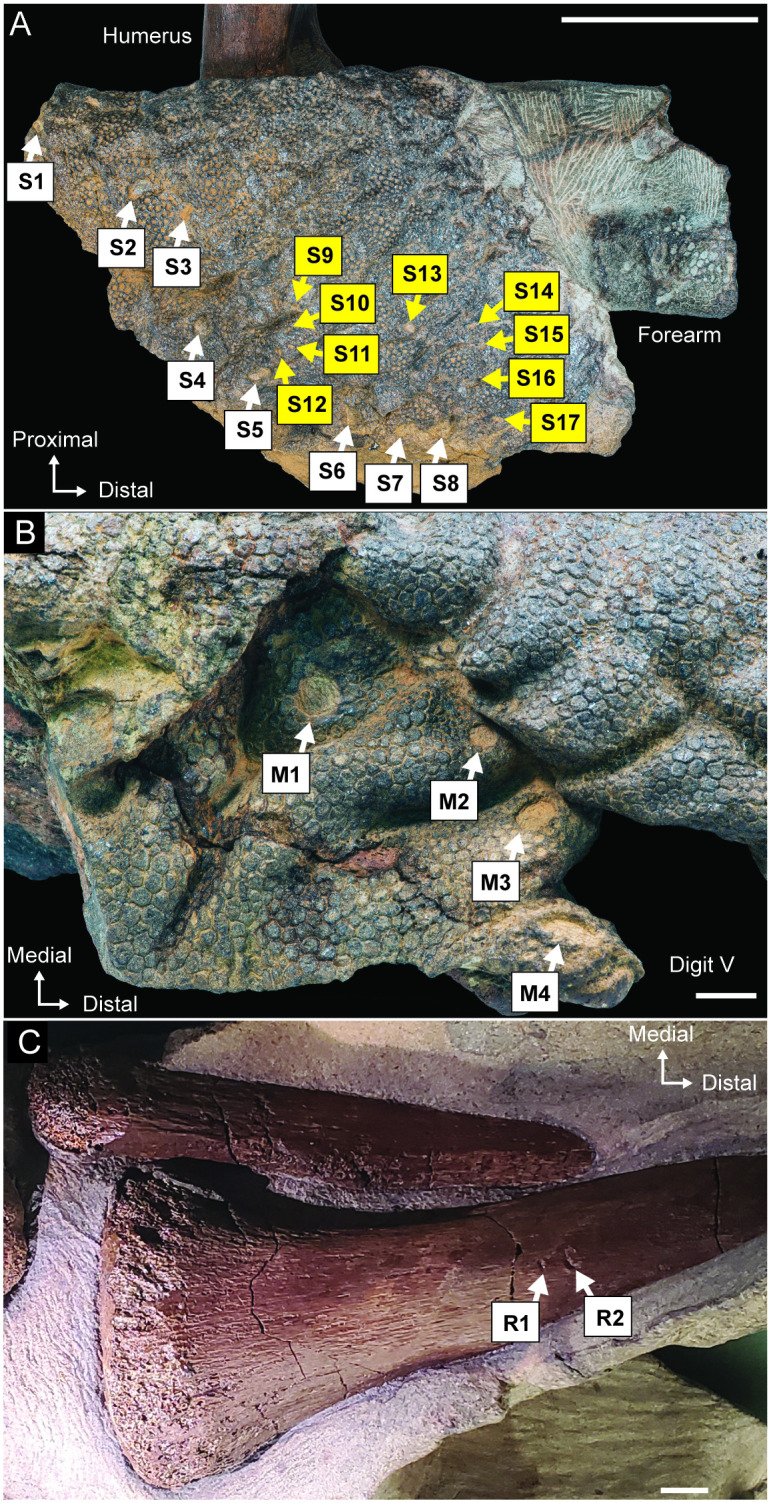
Locations of measured feeding traces on NDGS 2000 provided in Table 1. (A) Right lateral view of right elbow region. Coloration of arrows corresponds with that defined in Fig 5. (B) Dorsal view of manual digit V. (C) Proximal portion of right radius in anterolateral view. Scale bar in A equals 10 cm, scale bars in all others equal 1 cm.

**Fig 8:**
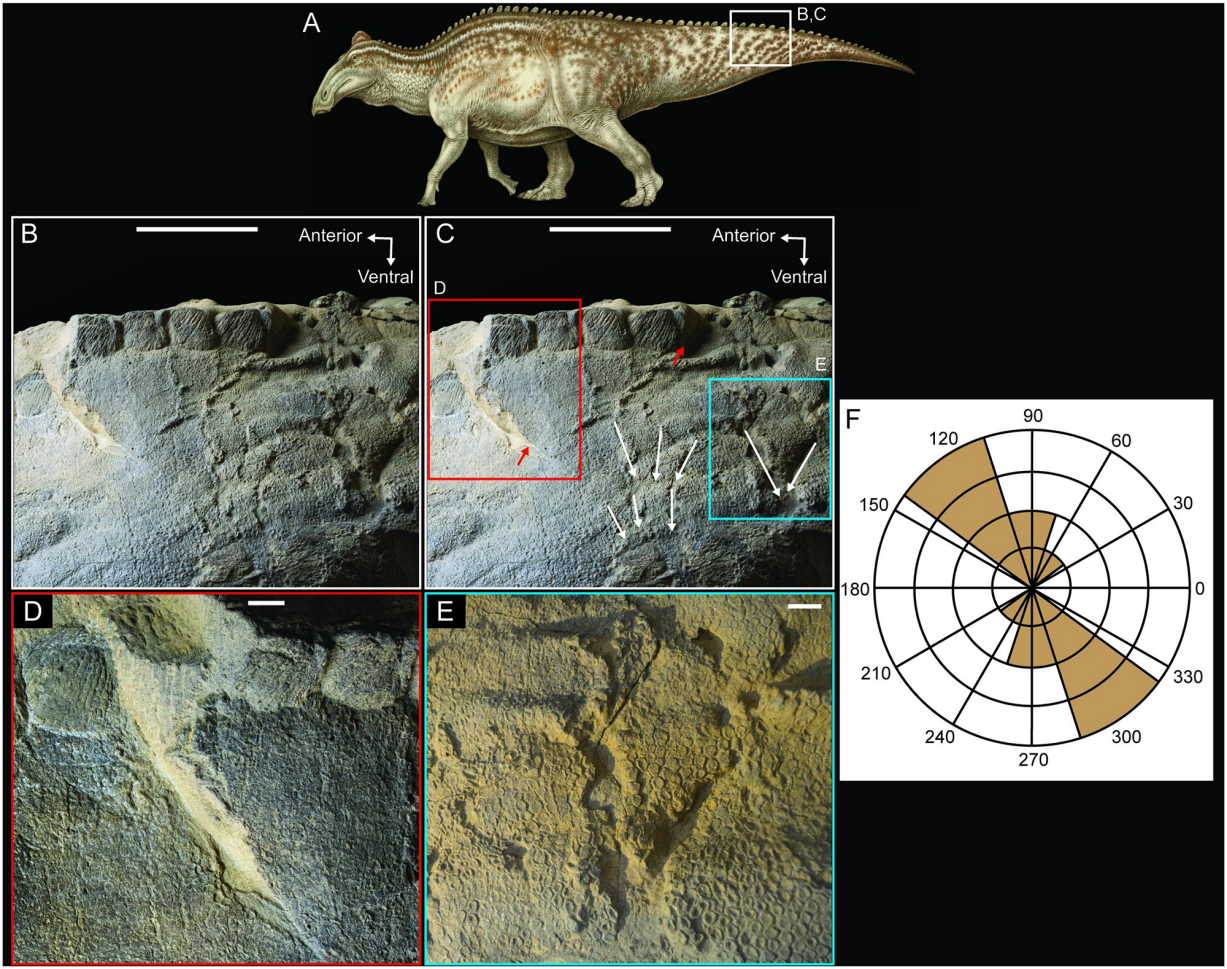
Soft tissue damage on the tail of NDGS 2000. (A) NDGS 2000 reconstruction in left lateral view showing the positions of the close-ups in B-E. (B) Close-up of the dorsal portion of the left lateral surface of the mid-caudal region of the tail providing an overview of the soft tissue injuries in this area. (C) Same view as in B, but with sets of furrows traced with white arrows, tears in the skin denoted with red arrows, and red and blue boxes indicating the positions of the close-up views shown in D and E, respectively. (D) Close-up view of posteroventrally oriented tear in the skin. (E) Close-up of one set of two furrows in the skin that converge ventrally, forming a ‘V-shaped’ tear in the skin. (F) Rose diagram of the orientations of the furrows in the left lateral surface of the tail measured relative to the dorsal margin of the tail. Zero degrees is positioned towards the distal end of the tail. Paleoart in (A) by Natee Puttapipat. Scale bars are 10 cm in B and C, and 1 cm in D and E.

Clusters of injuries are currently identified in three different locations on NDGS 2000: 1) the humerus, radius, and the soft tissues around the right elbow (Figs 5D–5F, 6B–6E, 7A and 7C); 2) in the soft tissues of the right manus around digit V (Figs 5C and 7B); and, 3) the left lateral surface of the tail, slightly more than halfway down its total preserved length (Fig 8). Specific counts and measurements of these pathologies are presented in Table 1 and Fig 7 provides a key to the locations of each measured trace on the skin flap near the right elbow, on the proximal portion of the right radius, and on the right manus. No evidence of healing is noted on any of the identified injuries. Preparation of NDGS 2000 is ongoing, and additional feeding traces may be discovered on currently unprepared areas (Fig 2) as work progresses.

**Table 1:** Dimensions of feeding traces on NDGS 2000.

Location	Trace Number	Max	Min
Right Manus	M1	8.7	7.9
Right Manus	M2	5.9	5.1
Right Manus	M3	8.5	8.1
Right Manus	M4	10.5	8.1
Right Forelimb Distal to Elbow	F1	5.3	3.5
Right Forelimb Distal to Elbow	F2	7.1	5.2
Right Forelimb Skin Flap	S1	9.5	5.8
Right Forelimb Skin Flap	S2	15.7	6.7
Right Forelimb Skin Flap	S3	8.3	3.8
Right Forelimb Skin Flap	S4	14.3	9.8
Right Forelimb Skin Flap	S5	14.3	8.5
Right Forelimb Skin Flap	S6	10.0	10.4
Right Forelimb Skin Flap	S7	8.0	6.2
Right Forelimb Skin Flap	S8	13.4	8.9
Right Forelimb Skin Flap	S9	4.9	3.0
Right Forelimb Skin Flap	S10	3.9	1.7
Right Forelimb Skin Flap	S11	4.8	2.0
Right Forelimb Skin Flap	S12	2.8	3.2
Right Forelimb Skin Flap	S13	6.3	4.6
Right Forelimb Skin Flap	S14	6.1	3.0
Right Forelimb Skin Flap	S15	5.0	3.5
Right Forelimb Skin Flap	S16	8.2	4.0
Right Forelimb Skin Flap	S17	5.4	3.3
Right Radius	R1	3.3	2.0
Right Radius	R2	2.4	1.4
Right Humerus	P1	6.0	2.7

Trace numbers correspond to labels in Fig 7. All measurements are in millimeters. Minimum widths were measured orthogonal to maximum widths. Abbreviations: max, maximum width of feeding trace; min, minimum width of feeding trace.

There are several punctures present on the dorsal surface of the manus around manual digit V, all of which are relatively circular (Fig 5C), potentially suggesting they were formed by conical rather than laterally compressed teeth. However, soft tissues can easily deform under a biting event and during any subsequent healing or decomposition, introducing uncertainty into extrapolating original tooth morphology [45, 46]. Additionally, even though the punctures are arranged in a roughly equidistantly spaced, arcing pattern, consistent with a set of serial punctures, without more obvious tearing it is impossible for us to completely exclude other taphonomic origins from contention, especially insect or other invertebrate borings [47]. That said, there seem to be additional punctures on the opposing side of the manus, which would further bolster the interpretation of these traces as a single, serial bite, but the damage is currently obscured by incomplete preparation and is only visible in CT scans. Several other additional obscured, potential punctures are also present on the distal, palmar surface of the manus, bridging digits II-IV, and again, they are only visible in the CT scans.

The marks on the more proximal region of the right forelimb are more diagnostic. There are numerous marks on the partially exposed right humerus and radius, as well as the surrounding skin. Unlike skin, bone provides a hard medium into which teeth can be impressed, and once tooth marks are generated, they do not undergo as much subsequent deformation in the absence of healing, given the rigid nature of the tissue. This results in a closer reflection of the impacting structure’s morphology in modified bone. The marks on the anterior surface of the humerus exhibit crushing damage consistent with impact trauma, which, when partnered with the roughly U-shaped cross section of the traces, are consistent with bite marks [19]. Of the traces on the humerus, a bisected pit and two serial hook scores are identifiable (Fig 6B–6E).

Hook scores have been associated with inertial feeding strategies, in which violent rolling or thrashing is used to dismember prey, resulting in abrupt changes in tooth trajectory during a single biting event [24–28]. The parallel curvature of the marks on the NDGS 2000 humerus suggest that they were formed at the same time, when multiple teeth contacted the bone during a single biting event. These types of traces are called serial scores, and these structures can sometimes aid in associating marks with specific actors by preserving tooth spacing and curvature along the toothrow [27].

The bisected tooth marks are diagnostic of crocodyliform actors who possess conical, carinated dentition [25–28]. These types of marks are formed when the prominent carina of a relatively unworn crocodyliform tooth leaves a distinct subscore through and notched margin around the indentation left by the apex of the tooth’s crown. These marks are expected in roughly 10% of all tooth marks left by extant crocodylians [25, 26], and have been used to positively associate traces with crocodyliform actors across members of the clade exhibiting this type of unspecialized dentition [27]. Two additional marks are present on the proximolateral surface of the radius (Fig 5E and 5F). The proximal of the two is a simple pit, but the second mark is another bisected pit, similar to that present on the humerus (Figs 5F versus 6D), with a short, slightly curved score extending from the medial margin. This combination of distinct pit and drag-out score is common in crocodylian-modified bones and is called a drag-snag [sensu 28]. These traces are all broadly consistent with any of the crocodyliforms present in the Hell Creek Formation, which includes *Brachychampsa montana*, *Borealosuchus sternbergii*, and possibly *Thoracosaurus neocesariensis* [18].

The injuries present on the soft tissue in close association with the humerus and radius are of a size and shape consistent with having formed during the same event that marked the bone surfaces. Prior to burial, the skin that originally covered the humerus was torn, inverted inside out, and pulled down the forelimb, partially degloving the arm and exposing the internal soft tissues and bones for further modification. There are at least seventeen punctures present in this inverted portion of skin (Fig 7A) and all are subrounded to slightly fusiform in shape. The larger punctures are concentrated along the torn margin of the skin, while the punctures that are farther from the torn margin are smaller in size (Fig 7A, Table 1). There are also at least two additional marks present in the soft tissue on the forearm, just distal to the degloved section of skin, which are rounded and lack the deformation caused by the degloving of the neighboring skin. It seems likely that the pits and scores on the humerus and radius and the punctures in the soft tissue on the posterolateral surface of the forearm were produced during the same biting event, they are broadly congruent in size and shape, but we cannot exclude the possibility of multiple trace makers at this time.

The remaining marks, present on the left lateral surface of the tail, are hard to interpret with confidence. All of these marks are punctures or furrows (i.e. penetrative, elongate marks), and the process of dragging teeth or claws through soft tissue distorts how well the injuries reflect the morphology of the impacting structures. The injuries to the tail are larger and often more elongate than those documented on the right forelimb, more widespread across the tissue, and range in a roughly dorsoventral direction (Fig 8B–8E). Some of the furrows converge in acute, ‘V-shaped’ angles, giving the marks a subparallel distribution as reflected in the Rose diagram (Fig 8F). A Rayleigh’s test of uniformity returns an R̅ of 0.8609 with p = 0.002, indicating a statistically significant mean direction within the sample. The spacing of these furrows suggests that a larger predator made them relative to the trace maker that modified the right forearm. The pliability of the soft tissue precludes completely ruling out teeth as the impacting structure, but the slightly converging, ‘V-shaped’ patterns (Fig 8C and 8E) suggest the possibility that flexible, clawed digits, rather than more rigidly fixed teeth, may have been responsible for these injuries. Any of the medium to large-sized carnivores, including crocodyliforms, larger deinonychosaurs (e.g., *Dakotaraptor steini*), and juvenile individuals of *Tyrannosaurus rex*, present in this ecosystem could be responsible for these traces [18, 48, 49].

More specific association of traces with trace makers in the hand and tail regions of this fossil seem unlikely with the evidence currently available. Actualistic observations of bite marks on skin have been complicated by the elastic deformation of the soft tissue during biting events and the warping of the initial trace by any subsequent healing or decomposition. This work has most heavily concentrated on human taphonomy, and the elastic nature of skin and underlying muscle has led to soft tissue bite mark analysis being largely abandoned in medico-legal circumstances, in which cases rely on the investigator’s ability to differentiate bite marks left by individuals of the same species (i.e., potential human suspects) [45]. However, differentiation between members of disparate species has been shown to be possible, owing to the expected initial differences in dentition, which can be reflected even in deformed soft tissue injuries [e.g., between humans and canids: 46]. While dinosaurian skin, and specifically that of *Edmontosaurus*, was substantially thicker and sturdier than human dermal tissues [5, 50], some pliability would still be expected during life and early stages of decomposition, adding a degree of uncertainty to how well injuries preserved in soft tissue might reflect the morphology of the marking tooth. At most, we can posit that the traces on the right manus and elbow are of comparable sizes and shapes, potentially suggesting the same actor was involved, while the damage to the tail suggests a larger trace maker. This implies that at least two different individuals interacted with the remains, but we cannot exclude the possibility that more individuals were involved in the partial consumption of NDGS 2000.

### Sediment analysis

**Fig 9:**
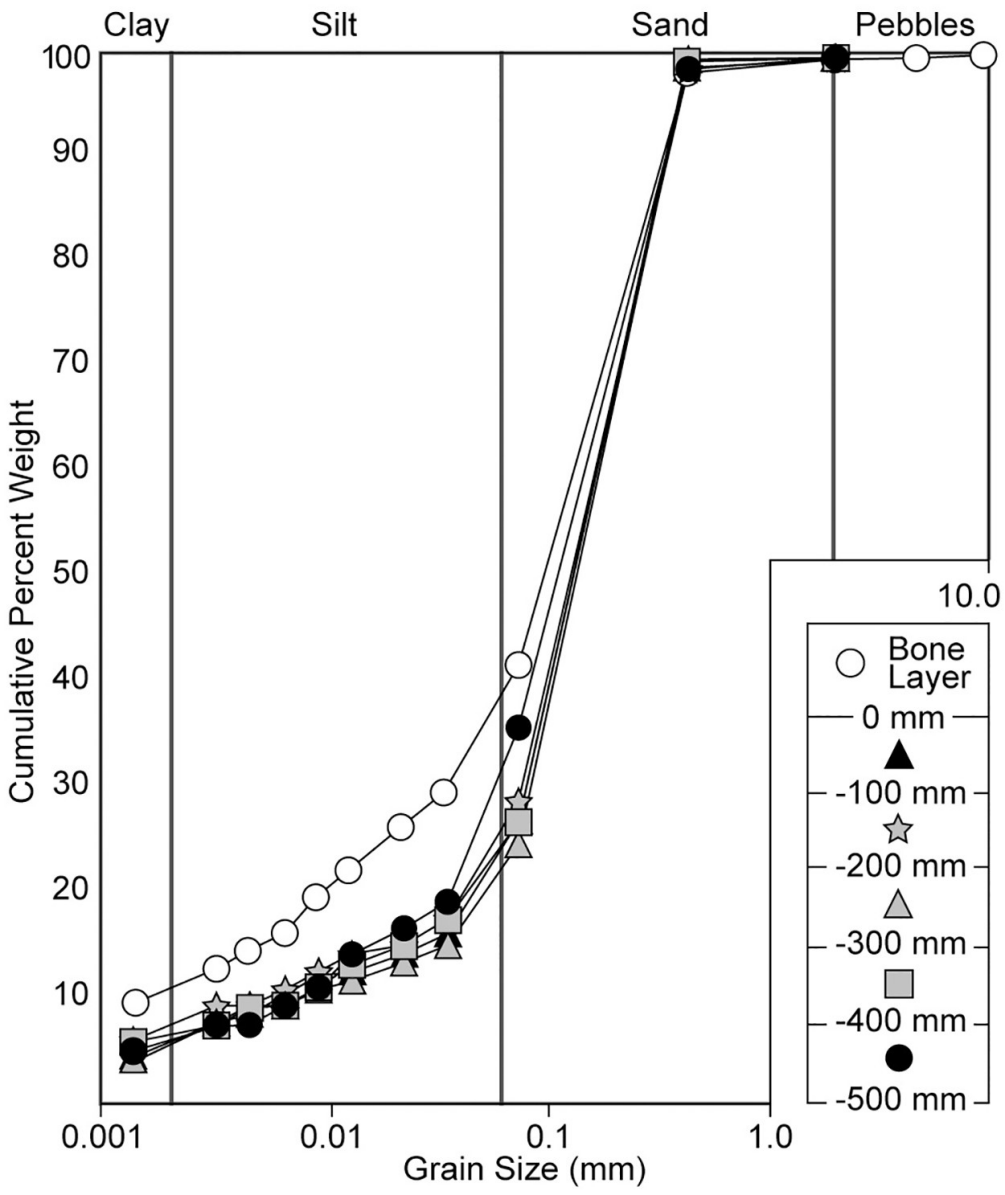
Grain size analysis of the rocks below and at the fossil layer preserving NDGS 2000.

Previous speculation that NDGS 2000 might have been scavenged by a crocodyliform relied not on identification of feeding traces, but instead upon the mummy’s close association with crocodyliform remains [7]. It was assumed that extreme aridity partnered with rapid burial during a flood would be necessary for the preservation of these soft tissues, and so the fossilization of the skin and the close proximity with a crocodyliform were both attributed to a sudden flooding event. To test that assumption, the sediment encasing NDGS 2000 was examined for any evidence of an increase in flow regime or for a rapid burial event at the level of the bone layer compared to the underlying sediment (Fig 9).

Prior paleoecological analysis of the site where NDGS 2000 was recovered suggests that NDGS 2000 was not deposited under arid conditions, but instead lived in a warm and humid environment, died in an area adjacent to persistent water sources, and was buried as part of a laterally accreting point-bar deposit [8]. The muddy sandstone deposited below NDGS 2000 is fairly consistent in composition in terms of the relative percentage of clay, silt, and sand. The sample of muddy sandstone collected from the fossil bearing horizon is slightly finer-grained than the underlying sediment, with higher percentages of clay and silt relative to sand (Fig 9, Table 2). Visual inspection of the sediment encasing NDGS 2000 revealed the absence of other lithologic evidence (e.g., rip up clasts) or sedimentary structures (e.g., scour marks) typically associated with higher energy depositional events like floods. Overall, the depositional environment is most consistent with a prograding sand bar [8] experiencing flow conditions that remained relatively constant or perhaps was slightly lower in energy when NDGS 2000 was buried compared to the conditions under which the underlying sediments were deposited. No sedimentological evidence of unusually arid conditions or rapid burial was discovered in this study, and in fact, the site seems to preserve a fluvial to deltaic, coastal forest [8, 14]. The partial crocodylian forearm preserved immediately below the skin of the calf region of the left hind limb of NDGS 2000 is currently the only skeletal remains recovered from the block that are not from the *Edmontosaurus* specimen. The association of NDGS 2000 with that crocodyliform forearm appears to be completely incidental and is best interpreted as confirming the presence of these taxa in the local environment.

**Table 2:** Grain size distribution of rocks encasing NDGS 2000.

Sample	Stratigraphic Position	% Clay	% Silt	% Sand
6	Bone Layer	10.8	31.0	58.1
5	0 cm to -100 mm	5.7	21.6	72.7
4	-100 mm to -200 mm	7.5	21.5	71.0
3	-200 mm to -300 mm	5.7	19.0	75.3
2	-300 mm to -400 mm	6.7	20.4	73.0
1	-400 mm to -500 mm	6.2	30.0	63.8

Note: Reported values are percent weight of the original 500-gram sample. Values may not sum to 100 percent owing to rounding of reported values.

## Discussion

The deflated appearance of NDGS 2000, in which skin persists in close proximity over the underlying bones, is reported in other dinosaur mummies [1, 2, 51] and a potential explanation for this type of preservation is documented in the forensic literature [52, 53]. These actualistic studies have determined that mummification of modern mammalian remains does not necessarily require an arid setting and can occur in relatively humid environments when remains have experienced incomplete scavenging (i.e. not complete skeletonization, sensu [47]) or other biotic interruption [52, 53]. Most vertebrate carnivores do not specifically seek out skin for consumption, and instead target the underlying muscles and viscera, which are generally more nutritious [60]. Larger carnivores may incidentally ingest large quantities of skin from smaller prey while accessing the tissue beneath, but smaller ones may instead create openings into the body cavity that allow active removal of internal tissues [52, 53]. Once the body cavity is compromised, these openings also permit smaller scavengers and decomposers to gain access to the interior of the remains. As the succession of scavengers and decomposers progresses, invertebrates join the larger macro- and mesopredators. These physically smaller, if more numerous decomposers are known to preferentially target injuries in order to access internal tissues [54]. Furthermore, injuries that penetrate the body wall also create an exit point from which the gases and liquids that are produced by the microbial breakdown of these same tissues during the active decay stage of decomposition can passively escape [47]. This type of carcass processing can somewhat unintuitively promote mummification of the skin because the absence of the internal tissues and their associated fluids and microbes result in slowed decomposition and more rapid drying of the skin and bones left behind [52, 53]. Mummified soft tissues preserved in this way can persist for months [55] during the transition from active decay to dry, skeletonized remains [sensu 47].

Incomplete carcass consumption, and the subsequent pattern of decomposition, is particularly relevant to understanding the mode of preservation in NDGS 2000 because this dinosaur mummy is not only deflated and desiccated, it also preserves direct evidence of carnivore interactions that allowed predators, scavengers, and decomposers access to the body cavity. Deep, raking furrows and punctures are present on the tail (Fig 8) and additional penetrative marks are present on the skin of the right manus and forelimb, the latter of which was partially degloved in the feeding process. While bite and claw marks on bone often preserve anatomical characteristics of the trace making structure, skin and other soft tissues deform more readily, especially in the event of subsequent healing or decomposition, a feature that confounds efforts to use injuries to identify specific sources of the subsequent damage in forensic [46] and paleontological [56] contexts, though major clades possibly can still be differentiated [46]. Fortunately, there are additional bite marks on the bones of this specimen, and those present on the right humerus and radius (Figs 5E, 5F and 6B–6E) are diagnostic of a crocodyliform trace maker [25–27].

The observed combination of desiccation and incomplete scavenging suggest that NDGS 2000 persisted on the landscape for some time prior to burial, which does not conform with previously proposed preservational pathways for dinosaurian mummies, which call for a largely undisturbed set of remains to be rapidly buried, essentially immediately, in the minutes to hours postmortem [1, 2, 4]. Under that model, disruption of the remains by predation, scavenging, or other biostratinomic processes would presumably inhibit preservation of soft tissues. That explanation may work well for mummies which exhibit three-dimensional preservation of possible internal organs with no evidence of desiccation [3]. However, when desiccation is noted, proposed explanations have grappled with the obvious conflict of preservational pressures [3, 11]. How would large dinosaurian remains, which were available food for any scavenger in the environment, persist for long enough to desiccate and deflate in an open landscape? Perhaps certain dinosaurian groups had particularly durable skin [5, 50]? Perhaps the remains were somehow made inaccessible after the animal’s death, or a mass death event or large-scale seasonal die-off occurred, providing a glut of resources for the meat eaters in an environment [1, 2, 10]? None of these explanations are particularly satisfying, especially in the absence of direct evidence supporting these extraordinary circumstances. Additionally, all of those explanations focus on the action of macropredators in the paleoenvironment, discounting the important role of mesopredators, small-bodied scavengers, invertebrates, and microorganisms, especially the microbial community that would have already been present both on and within the dinosaur when it died [57]. These physically smaller contributors to decomposition and fossilization processes have an outsized effect on decay rates and modes [58–60], and their hypothetical absence from the preservational history of any fossil is more difficult to explain away.

The state of preservation, documented injuries, and depositional environment of NDGS 2000 suggest a preservational pathway outside of the previous explanations of dinosaurian “mummification.” The remains were not left undisturbed during the decomposition and desiccation process; instead, they exhibit obvious injuries consistent with at least two different large carnivores interacting with the remains. The skin of the dinosaur’s limbs and tail are deflated, with the overlying tubercules and dermis preserved in exquisite detail, including biomolecules, and the underlying bones still articulated, but no other internal soft tissues are present during physical sampling or CT imaging, and no biomolecules are noted in the infilling sediments [13, 43, 44]. Sedimentological evidence suggests that the background sedimentation rate where this animal died was already low, and if anything, it slowed further, prolonging the time the carcass was exposed on the landscape. Under the previous explanations of dinosaurian “mummy” preservation, this fossil should have never formed.

**Fig 10:**
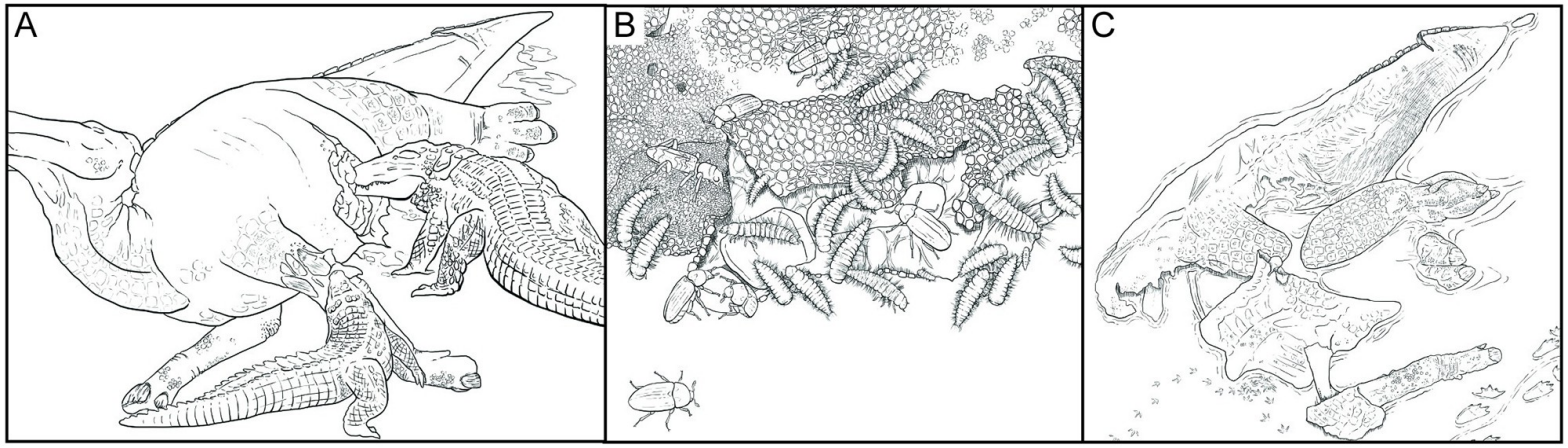
Proposed soft tissue preservational pathway based on examination of NDGS 2000 (*Edmontosaurus* sp.). (A) Incomplete predation and/or scavenging of the carcass creates openings in the body wall through which fluids and gasses can escape. (B) Invertebrates and microbes use those openings to access the internal tissues. (C) Removal of internal soft tissues and drainage of fluids and gasses associated with decomposition allows the deflated skin and other dermal tissues to desiccate and drape over the underlying bones. This process facilitates longer-term persistence of the skin and other resistant soft tissues until eventual burial and fossilization. Paleoart by Becky Barnes.

And yet, the processes that affected this *Edmontosaurus* are very consistent with pathways to natural mummification seen in actualistic research [52, 53]. Injuries inflicted during incomplete predation and scavenging would have allowed active and passive removal of the internal tissues and associated microbes, resulting in a largely deflated set of remains (Fig 10). Drying of the overlying skin could have progressed for weeks or months, until sediment from the adjacent river buried the remains. Therefore, early stage biostratinomic alteration of this *Edmontosaurus* actually lengthened the window for preservation of the skin as associated dermal tissues, an explanation for a set of observations that stand in stark contrast to previous explanations of natural mummification. These findings suggest that macro-scale soft tissue preservation did not necessarily require incredibly rapid burial, unusually hostile chemistry, immediate immersion into an anoxic environment, or fortuitous convergences of unlikely events to form. NDGS 2000 highlights a previously unexplored pathway for entraining soft tissues in the fossil record that utilizes typical biostratinomic processes elucidated from actualistic studies, eliminating the need to appeal to exceptional environmental or depositional events to explain the formation of dinosaur mummies [6]. The recognition of this pathway for soft tissue preservation through relatively common taphonomic processes helps to explain why fossilized skin, though still uncommon, is not exceptionally rare in the dinosaur fossil record, especially for the abundant hadrosauroids [39]. Additionally, given that the preservation of dinosaur mummies is here shown not to be dependent on rapid burial conditions in all cases, the discovery of a mummified specimen should not alone be used to infer the deposit represents a rapid burial event [6, 7]. Lastly, applying the wealth of actualistic research performed in modern forensic contexts to paleontological questions opens up new avenues of taphonomic research, including the possibility of more common soft tissue preservation outside of Konservat-Lagerstätten contexts than previously expected or understood.

## Conclusions

Previous attempts to characterize the preservational pathway of dinosaurian mummies have been confounded by the seemingly contradictory taphonomic processes of rapid burial and desiccation. Processes that would disturb or degrade the integrity of the remains, such as predation and scavenging, have been viewed as incompatible with long-term preservation of soft tissues. Research on the *Edmontosaurus* mummy NDGS 2000 suggests that this confusion is driven, at least in part, by attempts to come up with a unified explanation for all natural mummies. Instead of a single mode of preservation explaining these diverse sets of remains, we propose the following pathways:

### Desiccation and deflation

Remains preserved in this way have been exposed in a terrestrial setting for extended periods of time (weeks to months). During this time, the body wall becomes compromised through the activities of predators, scavengers, and decomposers including small-bodied invertebrates (Fig 10A and 10B). The ensuing openings provide a pathway for gases and fluids associated with microbial activity and the decomposition of internal organs and tissues to escape, effectively draining the remains until largely only dermal and skeletal tissues remain (Fig 10C). Once initiated, deflation and draining can continue during prolonged subaerial exposure or shallow burial. These processes result in a mummy that has a deflated appearance, with skin and associated dermal structures draped closely over the underlying bone. Internal organs and tissues are absent [47, 53, 54]. This preservational pathway is well-established by actualistic, forensic research, but has not previously been suggested in a paleontological context. NDGS 2000, a mummified example of *Edmontosaurus*, as interpreted in this study is an example of this mode of preservation.

Desiccation and deflation seem, based on existing publications, to be an exceedingly common (if not the most common) pathway for dinosaurian mummies to form [e.g. 1, 5, 51, 61–63]. The commonplace processes leading to this longer-term preservation of specifically dermal tissues may, beyond the durability of hadrosaurian skin itself, explain the unexpected frequency of its preservation [64, 65]. Similarly, the comparative paucity of facial skin [12, 66] may also be explained by this pathway, as predators, scavengers, and decomposers regularly target natural body openings, such as the mouth and eyes, first to gain access to internal soft tissues [67–69].

### Rapid burial

Rapid burial (defined here as ranging from the perimortem interval to a few days postmortem) removes remains from surficial taphonomic processes, including predation and scavenging. Encasement also limits access to oxygen, slowing microbial activity. These types of mummies are proposed to be preserved in three dimensions, potentially with internal organs as well as dermal tissues in place. However, pinpointing examples of this most-frequently cited mode of natural mummification is not without complications. A *Brachylophosaurus* mummy GPDM 115 has been cited as a classic example of this mode of preservation, with its largely intact skin and even gut contents and parasites [3, 70]. However, analyses of the body’s contents revealed conflicting results. No obvious internal organs were observed, leading to the suggestion that some combination of bacterial, enzymatic, and chemical processes might have broken down the non-dermal soft tissues [70]. The increased presence of clay within the body cavity relative to that in the encasing sediment further suggests that the remains were not buried intact, but instead that the body wall seemed to have been breached prior to final burial [3]. No obvious examples of predation, scavenging, or invertebrate-mediated damage were identified in GPDM 115, but natural orifices or an otherwise compromised body wall could have allowed liquified internal tissues to partially drain [3, 9]. Further work is required to assess the preservational pathway of this mummy, but for the moment, the specimen seems to potentially inhabit a middle area in the sliding scale between traditionally recognized rapid burial preservation and our proposed combination of desiccation and deflation, described above.

### Aqueous anoxia

Remains deposited in anoxic (no oxygen) to hypoxic (low oxygen) water experience limited to no scavenging and depressed microbial activity. This can take place whenever decomposition of deposited organics consumes most of the available oxygen, both in shallow bodies of water and at depth, when density stratification and stagnated circulation can result in anoxic deeper water underlying shallower, oxygenated water [71]. Burial rate can be variable under these conditions, and even in the absence of rapid sedimentation, remains can persist in anoxic or hypoxic waters for thousands of years prior to final burial [e.g., 72]. Additionally, aqueous anoxia itself can promote the mineralization of soft tissues, contributing directly to the fossilization process [e.g. phosphatization 71, 73]. This preservational mode is exemplified by TMP 2011.033.0001, a mummified specimen of the ankylosaur *Borealopelta* [74].

It is likely the pathways outlined above do not represent a comprehensive list of all possible processes that might result in the initial entombment of soft tissues leading to the formation of a ‘mummy.’ Nor are they meant to. There are numerous other types of soft tissue preservation that are not typically folded into the broad concept of mummification, including skin impressions occurring as negative molds or infilled casts [e.g. 75–80], frozen remains [e.g. 81, 82], and assemblage-wide patterns of fossilization within the context of Konservat-Lagerstätten, which themselves include a diversity of chemical and microbial pathways to disparate modes of preservation [e.g. 83–86]. Attempting to come up with a single explanation for the preservation of soft tissue in isolated remains presented paleontologists with contradictory evidence. In recognizing multiple pathways to form fossil ‘mummies,’ the seeming conflict can be settled in ways that do not require a spectacularly unlikely convergence of events and instead align with our existing understanding of more commonplace taphonomic processes.
